# Targeting Protein–Protein Interactions in the HIF System

**DOI:** 10.1002/cmdc.201600012

**Published:** 2016-03-21

**Authors:** Sarah E. Wilkins, Martine I. Abboud, Rebecca L. Hancock, Christopher J. Schofield

**Affiliations:** ^1^ Chemistry Research LaboratoryUniversity of Oxford12 Mansfield RoadOxfordOX1 3TAUK

**Keywords:** HIF, hydroxylation, hypoxia, inhibitors, oxygenases, protein–protein interactions

## Abstract

Animals respond to chronic hypoxia by increasing the levels of a transcription factor known as the hypoxia‐inducible factor (HIF). HIF upregulates multiple genes, the products of which work to ameliorate the effects of limited oxygen at cellular and systemic levels. Hypoxia sensing by the HIF system involves hydroxylase‐catalysed post‐translational modifications of the HIF α‐subunits, which 1) signal for degradation of HIF‐α and 2) limit binding of HIF to transcriptional coactivator proteins. Because the hypoxic response is relevant to multiple disease states, therapeutic manipulation of the HIF‐mediated response has considerable medicinal potential. In addition to modulation of catalysis by the HIF hydroxylases, the HIF system manifests other possibilities for therapeutic intervention involving protein–protein and protein–nucleic acid interactions. Recent advances in our understanding of the structural biology and biochemistry of the HIF system are facilitating medicinal chemistry efforts. Herein we give an overview of the HIF system, focusing on structural knowledge of protein–protein interactions and how this might be used to modulate the hypoxic response for therapeutic benefit.

## 1 Introduction

The chronic response to hypoxia (limited oxygen availability) in humans and other animals is substantially mediated by the α,β‐heterodimeric hypoxia‐inducible factor (HIF) transcription factor.^1^ HIF‐α protein levels are increased in hypoxia; it travels to the nucleus, dimerises with HIF‐β and binds to hypoxia response elements (HREs) in the promoter regions of HIF target genes. Together with transcriptional coactivator proteins, HIF promotes the context‐dependent expression of multiple genes that work to counteract the effects of hypoxia at a cellular, and subsequently, systemic level.^2^ Although there are other mechanisms of hypoxic adaptation, including those acting on a shorter time‐scale than the HIF system, the extent of the effects of the HIF system has led HIF to be characterised as a master regulator of the hypoxic response.

Many HIF target genes are of medical importance, especially in relation to cancer and ischaemic diseases. HIF target genes include those encoding erythropoietin (EPO) and vascular endothelial growth factor (VEGF), which induce the production of red blood cells and blood vessels, respectively, as well as many other proteins involved in metabolic and physiological adaptations to hypoxia. Modulation of the HIF system for therapeutic benefit is hence of considerable interest. Major efforts to date have focused on 1) the upregulation of HIF target genes (e.g., *epo*) for the treatment of anaemia and 2) the inhibition of HIF transcriptional activity as a cancer therapy. However, multiple other therapeutic applications of the HIF system can be envisaged, such as in wound healing or for the treatment of stroke. Further, because HIF is a pleiotropic transcription factor that is rapidly and efficiently induced by a gaseous small molecule, it is an attractive model system for basic studies on the control of gene expression.

The regulation of protein–protein interactions by oxygen‐dependent post‐translational modifications is central to the hypoxia‐sensing capacity of the HIF system. Crucially, it has been found that hydroxylation of HIF α‐subunits signals for their proteolytic degradation and regulates the transcriptional activity of HIF.^3^ The discovery of these modifications and the hydroxylases that catalyse them has opened up a new vista in oxygen‐dependent signalling, the relevance of which extends far beyond the HIF system. However, other protein–protein and protein–nucleic acid interactions play central roles in the HIF system and offer therapeutic possibilities. The purpose of this review is to give an overview of the HIF system, focusing on knowledge of the oligomeric interactions involved, and outlining how this knowledge might be exploited for therapeutic benefit.

### 1.1 The HIF transcription factors

**Figure 1:**
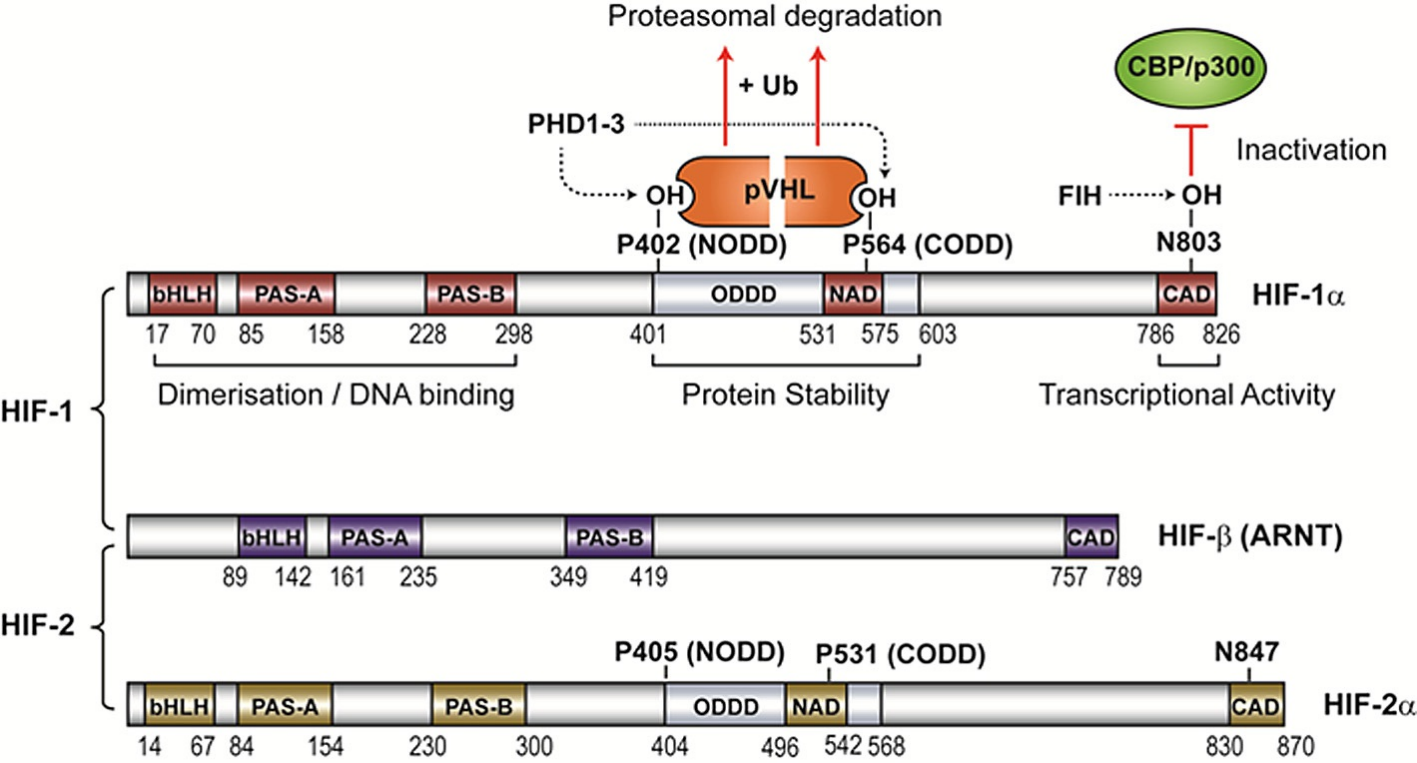
Functional domains in HIF transcription factors. HIF‐1α, HIF‐2α, and HIF‐β (also known as the aryl hydrocarbon nuclear receptor translocator, ARNT) are bHLH–PAS proteins; each contains a bHLH (basic helix‐loop‐helix) motif, two PAS (Per‐ARNT‐Sim) domains (PAS‐A and PAS‐B) and a C‐terminal transcriptional activation domain (CAD). HIF α‐subunits contain a second transcriptional activation domain (NAD), as well as N‐ and C‐terminal oxygen‐dependent degradation domains (NODD and CODD, respectively). The stability and transcriptional activity of the α‐subunits are influenced by protein–protein interactions with pVHL (von Hippel Lindau E3 ubiquitin ligase complex) and CBP/p300 transcriptional coactivator proteins. These interactions are regulated by the post‐translational hydroxylation of specific prolyl and asparaginyl residues in HIF α‐subunits, as catalysed by the PHDs and FIH, respectively. (Note: dotted lines correspond to oxygen‐dependent modifications.)

Active HIF transcription factors are comprised of an oxygen‐regulated α‐subunit and a constitutive β‐subunit.^4^ In humans there are three HIF‐α isoforms, of which HIF‐1α and HIF‐2α are the best characterised; together with HIF‐β (also known as ARNT) they form active transcription factors termed HIF‐1 and HIF‐2, respectively (Figure 1). HIF‐1 and HIF‐2 are closely related, but upregulate distinct (and sometimes overlapping) sets of genes in hypoxia.^5^ HIF α‐ and β‐subunits belong to the bHLH/PAS (basic helix‐loop‐helix/Per‐ARNT‐Sim homology) family of transcription factors (Figure 1).^4^ The bHLH and PAS domains mediate α,β‐dimerisation and DNA binding, while N‐ and C‐terminal transcriptional activation domains (NAD and CAD, respectively) recruit coactivator proteins to form active transcriptional complexes on DNA.^6^ HIF α‐subunits also contain an oxygen‐dependent degradation (ODD) domain, the hydroxylation of which renders them labile in oxygenated conditions.^7^

### 1.2 Regulation of HIF‐α by hydroxylation

The HIF α‐subunits have a short half‐life in normoxia due to their rapid turnover by the ubiquitin‐proteasome system.^7^, ^8^ An E3 ubiquitin ligase complex composed of Elongin C, Elongin B and the von Hippel Lindau tumour suppressor protein (pVHL) catalyses the poly‐ubiquitination of lysine residues that target HIF‐α for degradation by the proteasome (Figure 1). pVHL is the substrate recognition component of this complex and binds directly to HIF‐α; this interaction is substantially promoted by the hydroxylation of two proline residues in HIF‐α (P402 and P564 in HIF‐1α), located within N‐ and C‐terminal oxygen‐dependent degradation domains (NODD and CODD, respectively).^9^ A single hydroxylation at either site (NODD or CODD) is sufficient to target HIF‐α to the pVHL ubiquitin ligase complex for degradation.^9^ In some cancers, particularly kidney tumours, pVHL is inactivated by mutations; the resultant upregulation of HIF‐α may serve to promote tumour growth.^10^

HIF prolyl hydroxylation is catalysed by a set of non‐haem iron‐ and 2‐oxoglutarate (2OG)‐dependent prolyl‐4‐hydroxylases (PHD1‐3, also known as EGLN1‐3).^11^ Various lines of evidence imply that the catalytic activity of these enzymes is decreased under conditions of sub‐optimal oxygen availability, leading to a decrease in hydroxylation of HIF‐α.^12^ Because HIF‐α is not recognised by pVHL in the absence of prolyl hydroxylation,^13^ HIF‐α accumulates in hypoxia. The catalytic mechanism of the PHDs likely proceeds via the consensus for 2OG‐dependent oxygenases, that is, binding of 2OG to the active site is followed by that of substrate and finally, oxygen (for review see Ref.^14^). However, kinetic evidence implies that, at least for PHD2, the reaction of the enzyme with oxygen is unusually slow.^15^ Although slow reaction with oxygen is not a prerequisite property for a cellular hypoxia sensor, it is proposed to be advantageous in such a role.

A second mechanism of HIF regulation involves hydroxylation of N803 in the HIF‐1α CAD (N847 in HIF‐2α) by the 2OG‐dependent oxygenase Factor Inhibiting HIF (FIH).^16^ In contrast to HIF prolyl hydroxylation, which ‘makes’ a protein–protein interaction, asparaginyl hydroxylation of the HIF‐α CAD ‘breaks’ interactions between HIF‐α and the transcriptional coactivator proteins CREB binding protein (CBP) and p300,^17^ which are required for transcriptional activation of most HIF target genes.^18^ Like the PHDs, FIH is inactivated in hypoxia, though it retains activity at lower oxygen tensions than the PHDs.12c, 12f, 19 Thus, the HIF‐α CAD is silenced under normal oxygen conditions and transcriptionally active in hypoxia.

## 2 Protein–Protein Interactions in the HIF System

### 2.1 HIF‐α,β dimerisation

**Figure 2:**
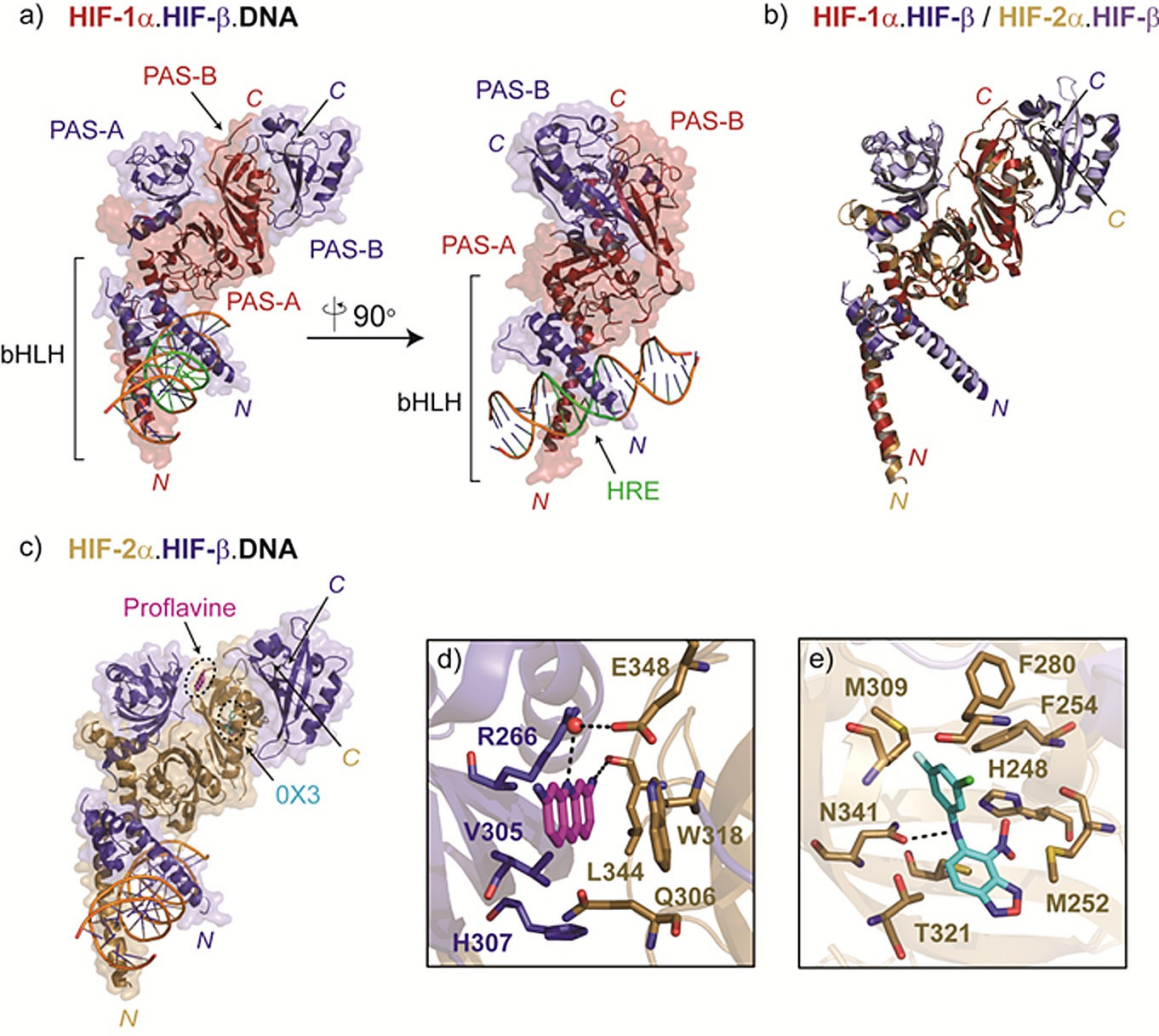
Architecture of HIF bHLH–PAS heterodimers. a) Two views from an X‐ray crystal structure of the HIF‐1α,β bHLH–PAS heterodimer in complex with DNA (PDB ID: 4ZPR). Domains in HIF α‐ and β‐subunits are indicated, and the HRE (hypoxia response element) is highlighted in green. b) Superimposed views from crystal structures of HIF‐1 and HIF‐2 bHLH–PAS complexes (PDB IDs: 4ZPR and 4ZPK, respectively). c) View from a crystal structure of the HIF‐2α,β bHLH–PAS heterodimer in complex with DNA (PDB ID: 4ZPK). Binding sites for the HIF dimerisation inhibitors 0X3 and proflavine are superimposed on the HIF‐2 heterodimer. Magnified views from crystal structures of HIF‐2 in complex with d) proflavine (magenta, PDB ID: 4ZPH) and e) 0X3 (cyan, PDB ID: 4ZQD) are shown in the adjacent panels. Important residues from HIF‐2α (gold) and HIF‐β (deep blue) that line the binding pockets are shown in stick representation. All images were generated using coordinates reported in Wu et al.^21^

Early studies involving deletion analyses of HIF proteins suggested that both the bHLH and PAS domains of HIF subunits contribute to heterodimerisation,^20^ an observation that is supported by recent crystallographic analyses of HRE‐bound HIF‐1 and HIF‐2 bHLH–PAS complexes.^21^ As illustrated in Figure 2 a, the dimerisation interface of the HIF‐1 complex is asymmetric; the bHLH, PAS‐A and PAS‐B domains of HIF‐1α pack together in a compact manner, whereas HIF‐β binds in an extended conformation, wrapping around the outer surface of HIF‐1α with relatively few intramolecular contacts. The HIF‐1α PAS‐B domain appears to be important in ‘scaffolding’ the complex, making contacts with the adjacent HIF‐1α PAS‐A domain, as well as both PAS domains in HIF‐β.

The helix‐loop‐helix regions of the HIF‐1 complex intertwine to form a stable helical bundle that straddles the DNA; two N‐terminal helices, one from each of the α‐ and β‐subunits, intercalate the DNA, binding the major groove on opposite sides of the double‐helix. HRE recognition is mediated by a cluster of basic residues on these N‐terminal helices, such that HIF‐1α and HIF‐β contact the 5′(AC) and 3′(GTG) ends of the HRE, respectively. The PAS‐A domain of HIF‐1α also contributes to DNA binding, with an extended loop that contacts the minor groove 6 bp downstream of the HRE.^21^, ^22^ Notably, the crystallographically observed conformation of the HIF‐2α,β dimer is near‐identical to that of the HIF‐1α,β complex (Figure 2 b) despite differences in amino acid sequence composition between HIF‐1α and HIF‐2α (66 % identical in the bHLH PAS region). The mode of HIF‐1 and HIF‐2 DNA binding is also highly similar in the reported structures (Figure 2 a and 2 c), rationalising the ability of HIF‐1 and HIF‐2 heterodimers to bind the same core HRE sequence.^21^, ^23^

### 2.2 HIF‐α ODD domain interactions: pVHL and the PHDs

**Figure 3:**
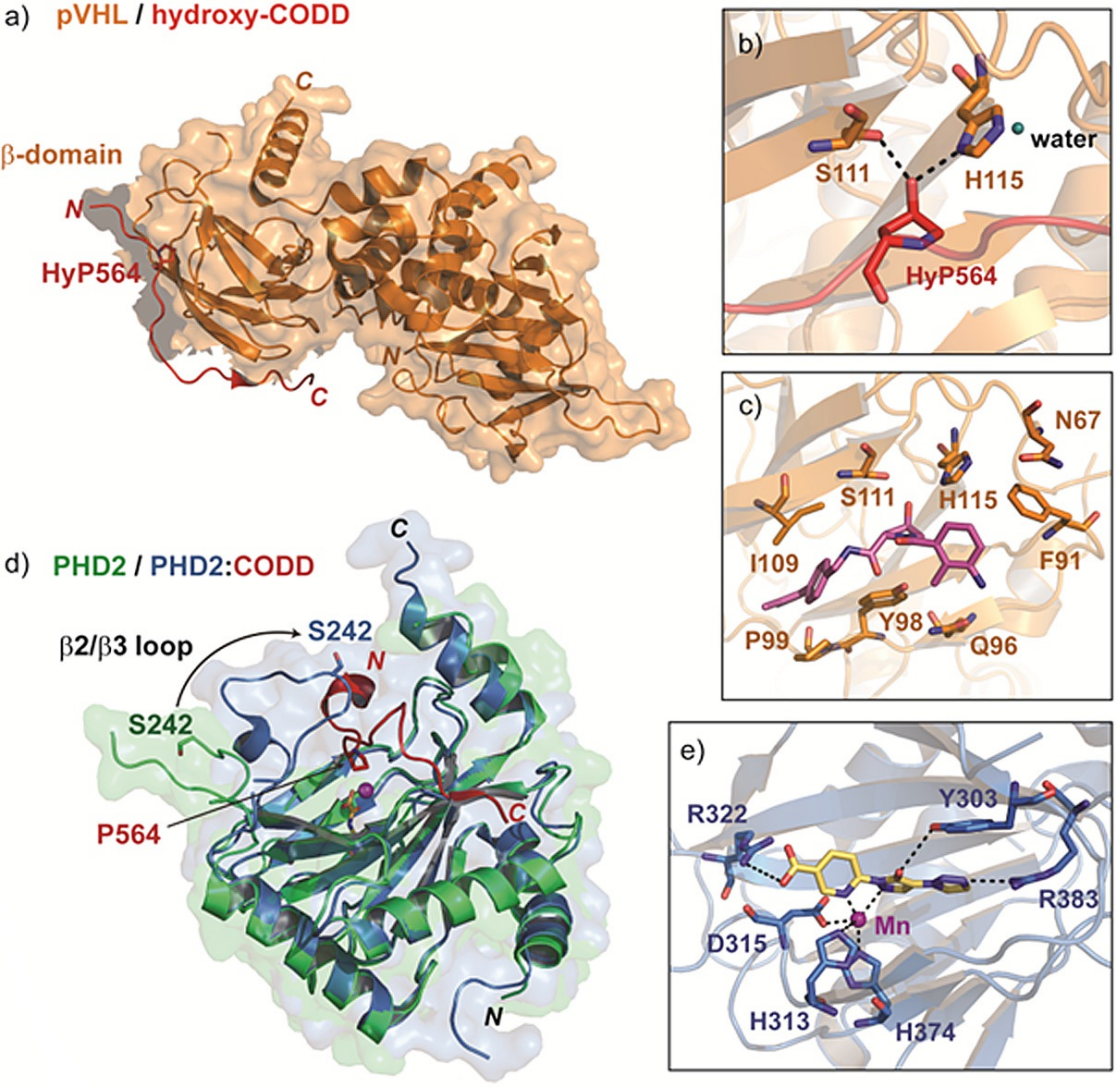
HIF‐1α CODD interactions with pVHL and PHD2. a) View from a crystal structure of pVHL in complex with a hydroxylated HIF‐1α CODD peptide (PDB ID: 1LQB 13). b) Magnified view from a) showing the orientation of HyP564 and its hydrogen bond interactions with residues in pVHL. c) View from a crystal structure of pVHL in complex with Ligand **51** 36 (purple), an inhibitor of the pVHL:HIF‐1α interaction. d) Superimposed views from X‐ray crystal structures of PHD2 alone (green, PDB ID: 2G1M 29) and in complex with a HIF‐1α CODD peptide (blue, PDB ID: 3HQR;^27^ CODD peptide is shown in red). e) Binding mode of a dihydropyrazole inhibitor (yellow) bound in the active site of PHD2 (PDB ID: 5A3U 37).

Crystal structures have been solved for pVHL in complex with a hydroxylated HIF‐1α CODD peptide.^13^, ^24^ The CODD peptide adopts an extended conformation when bound to the surface of a β‐sheet in the N‐terminal β‐domain of pVHL (Figure 3 a). Strikingly, a large number of tumour‐associated VHL mutations map to this interface.^25^ HIF‐1α is held in place through extensive backbone and side‐chain hydrogen bonds with pVHL, limiting its conformational flexibility. The C4 hydroxyproline residue in HIF‐1α (HyP564) is almost entirely buried at the interface. HyP564 is positioned to hydrogen bond with H115 and S111 in pVHL, rationalising the strict requirement for pVHL binding to a hydroxylated proline residue (Figure 3 b). Notably, the conformation of the proline ring appears to be an important determinant of pVHL binding, as is the C4 *trans* stereochemistry of the hydroxy group.^26^ The affinity of VHL for hydroxylated versus non‐hydroxylated CODD differs by almost three orders of magnitude, leading to the proposal that HIF‐α prolyl hydroxylation has a switch‐like effect on HIF signalling.^13^

As with binding to pVHL, the conformation of the target proline residue is important for HIF binding to the PHDs, as shown by work with PHD2.^26^ The non‐hydroxylated CODD proline adopts the C4 *endo* conformation when bound to PHD2; on the basis of crystallographic analysis, this conformation is proposed to be required for the productive reaction of a Fe^IV^=O intermediate with the C4 *trans* prolyl hydrogen atom.^27^ NMR and other biophysical studies reveal that binding of the HIF‐α ODDs to the PHDs involves substantial induced fit mechanisms, in particular involving a mobile loop region located between the β2/β3 strands of PHD2 at the C‐terminal region (Figure 3 d).^28^ The combined structural results imply that in the absence of HIF‐α ODD substrate, the β2/β3 loop is mobile and can be oriented away from the active site.^29^ On binding of a CODD peptide, the β2/β3 loop folds to entirely enclose the hydroxylation motif (LA*P*YIP).^28^ The importance of conformational changes in PHD catalysis is highlighted by biophysical analysis of a homologue of the human PHDs from *Pseudomonas* spp.^30^ Studies on the *Pseudomonas* hydroxylase (pPHD) in complex with its intact Elongation Factor‐Tu substrate reveal major conformational changes in both pPHD and EF‐Tu, which may be reflected in analyses of the intact PHDs and varied large HIF‐α fragments.^27^ The NODD is proposed to bind to the PHDs in a similar manner to CODD, though details of the interaction must be different. Mutational analyses indicate that that L574, located 10 residues downstream of the HIF‐1α CODD hydroxyproline, is an important determinant of PHD2 binding;^31^ however, a leucine is not present at the equivalent (+10) position relative to P402 in NODD. As yet, there are no structures for PHD:NODD complexes.

It is important to emphasise that pVHL‐ and hypoxia‐independent mechanisms of HIF (de)stabilisation occur. Antibody‐based studies indicate that, at least in some cases, HIF‐α is upregulated in cancer cells but still undergoes prolyl hydroxylation.^32^ Although these observations could be due to impaired pVHL function, it is likely that other factors can limit HIF‐α degradation. Several reports have linked heat shock proteins to HIF stability, with both HSP90 and HSP70 being reported to interact with HIF‐α.^33^ HSP90 is proposed to bind to HIF‐α in the cytoplasm and protect it from oxygen‐independent degradation.^34^ Displacement of HIF‐α from HSP90 by small‐molecule inhibitors (e.g., geldanamycin) enables binding of RACK1 (receptor of activated protein C kinase 1), which recruits the ubiquitin ligase machinery and potentiates HIF‐α degradation.^35^ HSP70 and the ubiquitin ligase CHIP (C‐terminal Hsp70 Interacting Protein) are reported to promote HIF‐1α, but not HIF‐2*α*, degradation, so blocking HIF‐1 activity.33b Recent interesting work has also identified a role for the MYND (N‐terminal Myeloid Nervy and DEAF‐1) zinc‐finger domain present in PHD2 homologues, which binds to a conserved motif in co‐chaperone proteins, including p23 of the HSP90 system. P23 is proposed to recruit PHD2 to the HSP90 machinery to facilitate hydroxylation (and degradation) of HIF‐1α.^38^

Multiple other interacting partners for the PHDs have been described, including the tumour suppressor protein LIMD1 (LIM domain containing protein 1), which simultaneously binds the PHDs and pVHL in a manner that promotes HIF‐α degradation.^39^ Many other interacting proteins have been reported to be PHD substrates on the basis of antibody and/or proteomic mass spectrometry analyses. The relevance of these interactions to the hypoxic response remains to be validated, though the findings do raise the possibility that competition for binding to the PHDs may be regulatory. Such competition is more established for FIH, which we focus on in this review (see^40^ for a review of non‐HIF PHD substrates).

### 2.3 HIF‐α CAD interactions: FIH and CBP/p300

**Figure 4:**
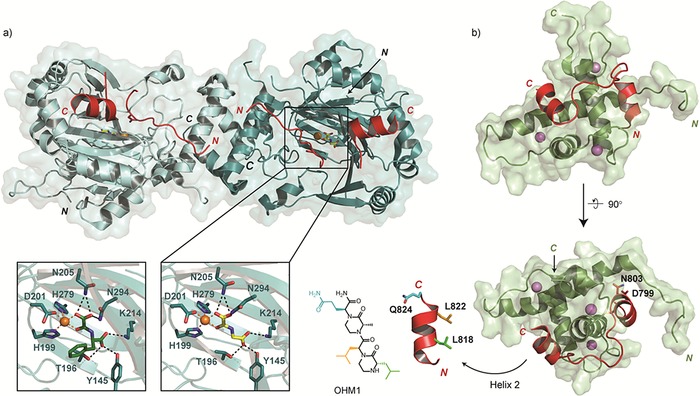
HIF‐1α CAD interactions with FIH and p300. a) View from a crystal structure of FIH (shown as a dimer, monomers coloured light and dark teal) in complex with the HIF‐1α CAD (red), Fe^II^ (orange) and 2OG (yellow, PDB ID: 1H2K 44). Panels underneath show a magnified image of the FIH active site bound to 2OG (right, PDB ID: 1H2K 44) or the FIH‐selective inhibitor N‐(carboxycarbonyl)‐d‐phenylalanine (NOFD, left, PDB ID: 1YCI 54). b) Two views from an NMR structure of the CH1 domain of p300 (green) in complex with a HIF‐1α CAD peptide (red, PDB ID: 1L3E 42a). Two helices are induced in HIF‐1α upon binding; a magnified image of Helix 2 (HIF‐1α residues 814–824) is shown underneath, alongside the oxopiperazine peptidomimetic inhibitor OHM1.^55^

CBP/p300 interact with both the NAD and CAD in HIF‐α,^41^ though only the latter of these interactions is known to be regulated by oxygen‐dependent hydroxylation and has been structurally characterised.^17^, ^42^ NMR structures of the CH1 domains of CBP and p300 in complex with HIF‐1α CAD peptides have been reported.^42^ In both cases, the four alpha helices that constitute the CH1 domain form a bundle that is stabilised by coordination with three Zn^2+^ ions. The CAD folds around the CH1 domain like a clamp, adopting two induced α‐helices that bind in an almost parallel arrangement on opposite faces of the CH1 domain (Figure 4 b). N803 in the HIF‐1α CAD is located on the N‐terminal helix (Helix 1) and is buried within the molecular interface. Hydroxylation at the *pro‐S* position of N803, which blocks HIF binding to CBP/p300, likely creates a direct steric clash with the backbone carbonyl of D799 in HIF‐1α, so disrupting the formation of this helix (Figure 4 b).^42^, ^43^ The tertiary structure of the HIF‐1α CAD when complexed with the CH1 domain of CBP/p300 is determined almost exclusively by intermolecular contacts; circular dichroism analyses indicate that the isolated HIF‐1α (and likely HIF‐2α) CAD is intrinsically disordered in solution.^42^

The available evidence indicates that FIH does not undergo such major conformational changes as the PHDs on substrate binding.^44^ When bound to FIH, the HIF‐1α CAD adopts an extended conformation that is less enclosed than that of CODD binding to PHD2.^27^, ^44^ Multiple hydrogen bonds are involved in binding the CAD to FIH, as well as hydrophobic interactions, including with a valine residue present immediately N‐terminal to N803 in the HIF‐1α CAD hydroxylation motif (CEV**N**AP); this valine forms a hydrophobic interaction with W296 of FIH, which is involved in an induced fit mechanism.^44^ The primary amide of N803 is positioned to form hydrogen bonds with conserved residues in FIH, notably Q239. The interaction of HIF‐1α with FIH is likely more complex beyond the immediate vicinity of the active site; a second binding site is observed in which the HIF‐α residues involved form an α‐helix, as observed for these same residues bound to the CH1 domain of CBP/p300 (Figure 4).^42^, ^44^

FIH is reported to interact with many proteins other than HIF‐α, mostly coming from the ankyrin repeat domain structural family (see^45^ for detailed reviews on FIH‐catalysed ankyrin repeat hydroxylation). There is strong cellular and biochemical evidence that many (but not all) of these are hydroxylation substrates for FIH. From a structural biology perspective, the finding that FIH accepts ankyrin repeat domain proteins was surprising, as they must undergo significant unfolding in order to bind productively at the active site.^46^ FIH‐catalysed ankyrin repeat hydroxylation occurs at conserved positions and not just on asparagine residues.^47^ Because some ankyrin substrates are of major biological importance (e.g., Notch^48^), there was interest in the possibility that FIH‐catalysed ankyrin repeat domain hydroxylation might have ‘switch‐like’ roles in cellular processes, although as yet there is no clear evidence for this. At least in some cases, hydroxylation stabilises the stereotypical ankyrin fold.^49^ Nonetheless, as the extent of hydroxylation is rarely complete,^50^ it would seem unlikely that ankyrin repeat domain hydroxylation plays a crucial structural role (in contrast to *trans*‐4‐prolyl hydroxylation which stabilises the collagen triple‐helix fold^51^). One possibility is that competition for FIH between the HIF‐α CAD and ankyrin repeat domains serves to modulate the HIF‐mediated hypoxic response such that it can function in different environments;48a, 52 another is that ankyrin repeat domain hydroxylation can provide a memory of hypoxic events.^53^ The discovery of FIH‐catalysed hydroxylation of multiple ARDs also raises the unanswered question of whether HIF‐α needs to be targeted to FIH. To enable this, it is possible that a targeting protein may bind to one monomer of the FIH dimer, so promoting binding of HIF‐α to the other.

It is important to appreciate that in addition to the regulatory oligomeric interactions described above (i.e., HIF:HRE, HIF:PHD/pVHL, HIF:FIH/CBP/p300), many other protein–protein interactions are involved in the regulation of HIF‐mediated transcription, as is likely the case for any pleiotropic transcription factor. Modulation of these complex and dynamic interactions, which may occur at transcriptional, RNA‐processing/splicing, translational and post‐translational levels, offers potential for control of the set of HIF target genes that are upregulated. Further, post‐translational modifications such as phosphorylation, acetylation and ubiquitylation are very likely to be important factors in HIF regulation. At present, we have a poor understanding of the role of these other interactions on the kinetics of HIF‐mediated transcription. HIF has been reported to interact with other transcription factors, notably Notch^56^ and NFκB,^57^ and is proposed to regulate, and be regulated by, multiple proteins involved in transcription/chromatin biology.^58^ In this regard, the Jumonji‐C histone demethylases are notable since they belong to the same structural subfamily of 2OG oxygenases as FIH.^59^

### 2.4 Protein–protein interactions involving HIF‐β

HIF‐β (ARNT) is a common dimerisation partner for members of the bHLH/PAS transcription factor family, including the aryl hydrocarbon receptor (AHR), which is involved in xenobiotic metabolism, and single‐minded proteins (SIM1‐2).^60^ Although there is some cross‐talk between the different signalling pathways involving HIF‐β, the available evidence is that the HIF‐mediated hypoxic response is not substantially limited by competition for HIF‐β.33a Nevertheless, this could occur in certain contexts. Further work is required on the interplay between HIF‐α/HIF‐β and other bHLH/PAS domain transcription factors, and how this leads to context‐dependent differences in HIF target gene regulation. Interestingly, a hypoxically upregulated splice variant of HIF‐3α known as IPAS (Inhibitory PAS Domain Protein) is proposed to compete with HIF‐β for binding to the other HIF α‐subunits and function as a negative regulator of HIF signalling.^61^ More recently, the PAS‐B domain of HIF‐β has been shown to interact directly with a number of coiled‐coil coactivator proteins, including thyroid hormone receptor interacting protein 230 (TRIP230).^62^ HIF‐β‐mediated recruitment of TRIP230, and potentially other coiled‐coil coactivators, may be important for transcription of some HIF target genes. As such, the development of small molecules that disrupt these interactions could provide an alternative means of HIF regulation.^63^

## 3 Inhibition of Protein–Protein Interactions in the HIF System

The HIF system is presently perceived to be an attractive pathway for pharmacological intervention. One reason for this is that the discovery of HIF and its regulatory elements was motivated by a physiology‐driven research approach, that is, to understand the underlying molecular mechanisms behind the hypoxia‐induced upregulation of EPO.^64^ A second, related reason is that there is strong evidence that modulation of the activity of a limited number of key players in the HIF system can have profound physiological consequences. Although the factors involved in context‐dependent regulation of the HIF system are incredibly complex, the ‘core’ hypoxic response is principally mediated by a relatively small number of players, that is, HIF, PHD2/VHL, FIH/CBP/p300. This situation was largely unanticipated; even after the discovery of the PHDs and FIH, we expected that other direct hypoxia sensors for the HIF system would be discovered. To date this has not been the case, although other oxygenases (e.g., histone demethylases) no doubt play a role in transcriptional regulation by HIF and might do so in a hypoxically regulated manner.59b, 59c, 65 A key early concern with respect to pharmacological intervention of the HIF system was that its pleiotropic nature might be a safety issue, for example, PHD inhibitors might promote tumour growth by promoting VEGF production. However, counter‐arguments include knowledge that major drugs do target transcriptional regulation,^66^ living at altitude does not apparently cause a significantly increased incidence of cancer,^67^ and that cobalt ions have been used for treatment of anaemia in a mechanism proposed to involve PHD inhibition.11b, 68 Perhaps the most important evidence is that PHD small‐molecule inhibitors are now in late‐stage clinical trials for the treatment of anaemia and are being considered for treatment of other hypoxia‐related conditions, including stroke and ischaemic diseases.^69^ Studies to date show, at a minimum, that it is possible to target the HIF system in the short term without serious (i.e. life—threatening) side effects. An increasing number of small molecules have been reported to modulate the HIF system by altering HIF mRNA levels, protein synthesis/stability, DNA binding and transactivation.^70^ Here we focus on molecules that interfere with key protein–protein interactions—specifically, binding of the HIF α‐subunit to HIF‐β, pVHL and CBP/p300.

Protein–protein interactions are often perceived to be difficult to target with small molecules. However, the biological effects of FIH‐ and PHD‐catalysed hydroxylation have been inspirational to efforts targeting protein–protein interactions, revealing how a very small modification, that is, addition of a single neutral oxygen atom, can have profound effects on protein–protein interactions.^13^, ^17^ Indeed, the role of HIF‐α prolyl hydroxylation has led to a new general approach to targeting proteins for degradation with small molecules.^71^ Such efforts are crucially informed by structural analyses, with a recent highlight being the report of structures for the HRE‐bound HIF‐α,β complexes.^21^

### 3.1 Inhibition of the PHDs and FIH

Targeting the PHDs via small‐molecule inhibition has been a major focus of pharmaceutical efforts on the HIF system to date. The vast majority of the reported PHD inhibitors, and all of those in clinical trials, likely work by binding the active site iron and competing with 2OG (Figure 3 e), though there are variations in binding modes, selectivity, and the extent to which they inhibit HIF‐α binding. Because descriptions of these approaches to PHD inhibition have been reviewed in detail elsewhere,^69^ we do not describe them here; instead we focus on the likely influence of protein–protein interactions on the role of the PHDs.

In our view, it is probably a mistake to think of the PHDs (and FIH) only as enzymes catalysing post‐translational modifications. It may well be that the stoichiometric protein–protein interaction between the PHDs and HIF‐α plays a role in the hypoxia response. At the very least, understanding the details of the PHD:HIF‐α interaction may be useful in optimising PHD inhibitors, for example, some inhibitors more efficiently displace HIF‐α from the PHDs than others.^37^ The development of substrate‐selective inhibitors is of particular interest given the distinct physiological roles of HIF‐1 and HIF‐2 target genes.5a, 72 Further, compounds that promote PHD activity are also of interest, in particular from a cancer pharmaceutical perspective. Such compounds might work by strengthening the PHD:HIF‐α protein–protein interaction or by promoting the rate of its reaction with oxygen. An alternative strategy for the latter would be to direct oxygen to the PHDs inside cells. In this regard, it is of interest that a mitochondrial respiration targeting compound has been recently reported to promote HIF‐α degradation, possibly by promoting PHD activity.^73^ Reducing agents, such as ascorbate, have also been found to promote PHD activity in vitro, as is the case for some other 2OG oxygenases, most famously the collagen prolyl hydroxylases.^74^ Although the effects of ascorbate are very unlikely to be selective for the PHDs, the cellular redox balance is likely to impact on the HIF system.^75^ The emerging structural data on PHD:HIF‐α interactions should also enable efforts in pharmaceutical modulation of PHD activity beyond simple inhibition by active site binding.

There has been much less work on FIH inhibition than the PHDs. This may be for several reasons, including: 1) because FIH is of less fundamental importance than the PHDs in the hypoxic response, 2) because the role of FIH is linked to the function of the pleiotropic transcriptional coactivators CBP/p300, and 3) because FIH has multiple other substrates/binding partners than the HIF‐α CAD (see above). Only a limited number of FIH inhibitors have been reported,^76^ though early work on FIH was important in that it demonstrated that selectivity for specific 2OG‐dependent oxygenases could be achieved.^54^ In the case of FIH and the PHDs, selectivity can be achieved by exploiting the smaller 2OG binding pocket present in the PHDs relative to FIH.^44^ Further, modulating the complex protein–protein interactions involved in the FIH/HIF‐α/ARD axis is of potential therapeutic interest, for example, blocking ARD, but not HIF‐α, binding to FIH should promote FIH‐mediated inactivation of HIF‐α activity, which might be of use from a cancer perspective.

### 3.2 Targeting the HIF‐α,β dimer

**Figure 5:**
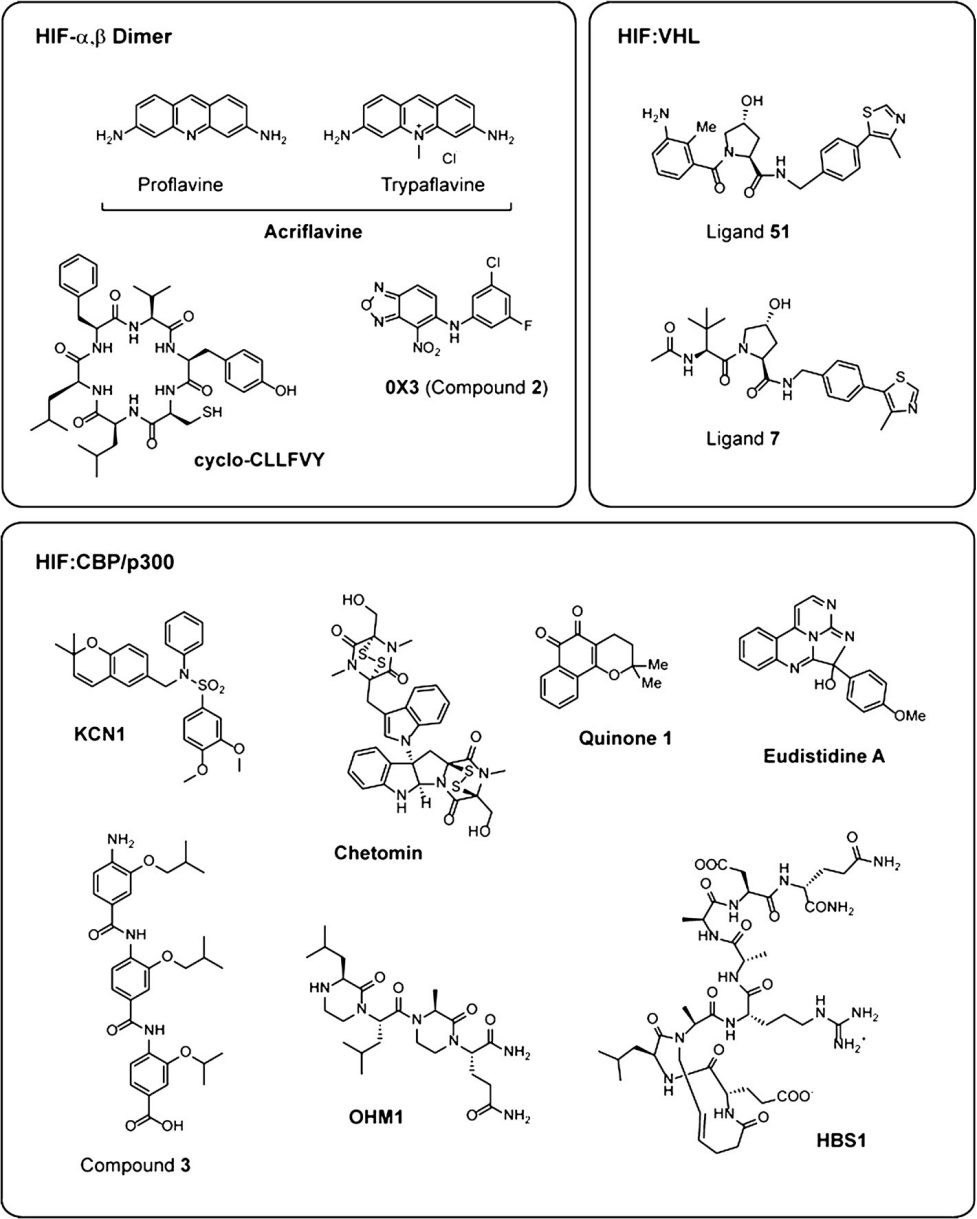
Small‐molecule inhibitors of HIF protein–protein interactions. A selection of compounds modulating HIF protein–protein interactions are shown, including those targeting 1) the HIF‐α,β dimer: acriflavine,^78^ 0X3,80c and cyclo‐CLLFVY;^81^ 2) HIF‐α:pVHL interactions: Ligand **51** 36 and Ligand **7**;^82^ 3) HIF‐α:CBP/p300 interactions: KCN1,^83^ Chetomin,^84^ Eudistidine A,^85^ Quinone **1** 86 and the peptidomimetic inhibitors OHM1,^55^ Compound **3**,^87^ and HBS1.^88^

Formation of the α,β‐HIF heterodimer is an apparently strict requirement for HIF to bind to DNA and promote transcription,^20^, ^77^ making this protein–protein interaction an appealing target for pharmaceutical intervention. One of the first drugs reported to modulate HIF dimerisation was acriflavine, which is reported to bind at the interface between the HIF‐α PAS‐B and HIF‐β PAS‐A domains and to destabilise the HIF‐α,β heterodimer.^78^ Acriflavine is used as a topical antiseptic and is a combination of two structurally related flavins, trypaflavine and proflavine (Figure 5), both of which reportedly bind to HIF‐1 and HIF‐2 with low nanomolar affinities in vitro (40 nm and 41 nm for HIF‐1 and HIF‐2, respectively).^21^ Recent crystallographic analyses reveal that proflavine makes contacts with residues R266 and V305 in HIF‐β, which are critical for maintaining the integrity of the interface with HIF‐α (Figure 2 c and 2 d).^21^ The binding site for trypaflavine is unknown, but given its structural similarity to proflavine, it is predicted to be comparable. Acriflavine inhibits HIF‐mediated transcription in cultured cells and tumour xenografts,^78^and has been shown to decrease tumour growth and vascularisation, likely in part through inhibition of HIF signalling.^79^

Several studies have explored a buried, largely hydrophobic cavity within the HIF‐2α PAS domain as a potential site for binding of allosteric inhibitors.^80^ This site was first revealed in crystallographic studies of the isolated PAS‐B domain of HIF‐2α,80d but is also evident in the recently reported structure of the intact bHLH–PAS domain.^21^ Work from Scheuermann et al. has shown that this pocket can accommodate a variety of bicyclic ligands, some of which perturb the formation of the active HIF‐2 heterodimer.80a, 80d The most potent of these compounds (0X3, Figure 5) binds to the isolated HIF‐2α PAS‐B domain in vitro with nanomolar affinity,80b, 80c and disrupts HIF‐2α/HIF‐β dimerisation and transcriptional activity at low micromolar concentrations in cultured cells.80c 0X3 and related bicyclic inhibitors are proposed to act via an ‘allosteric’ mechanism, inducing conformational changes in the isolated HIF‐2α PAS‐B domain such that it is no longer able to bind HIF‐β PAS‐B.80c Crystallographic analyses of 0X3 bound productively to the intact HIF‐2 bHLH–PAS dimer (Figure 2 c and 2 e) reveal few structural differences between the apo and liganded complexes,^21^ suggesting it is the dynamics rather than the structure of the PAS‐B domain that is altered by ligand binding;^89^ if correct, this proposal raises interesting possibilities for the fine‐tuning of HIF transcriptional activity. Interestingly, co‐ and chromatin‐immunoprecipitation experiments reveal that 0X3 disrupts the formation and HRE‐binding of the HIF‐2‐α,β heterodimer, with little effect on HIF‐1.80c These observations can be rationalised by structural comparison of the HIF‐1α and HIF‐2α PAS‐B domains; although a similar cavity exists in HIF‐1α, it is considerably smaller and differs in the composition of residues that line the binding‐pocket.^21^, 80c The discovery of substantial cavities in HIF‐1α and HIF‐2α raises the intriguing possibility that HIF is regulated by endogenous ligands, though as yet, none have been discovered. Nevertheless, these pockets appear to be ‘hot‐spots’ for inhibitor development. Cardoso et al. have shown that the allosteric cavity in HIF‐1α is amenable to small‐molecule inhibition.^90^ More recently, a cyclic peptide inhibitor (cyclo‐CLLFVY, Figure 5) that selectively inhibits HIF‐1α,β dimerisation has been described. This peptide is reported to decrease HIF‐1‐mediated activity in a variety of cell lines, apparently without affecting the function of the closely related HIF‐2 isoform.^81^

Taken together, the combined studies suggest that the HIF‐α PAS‐B domain is an interesting target for the development of inhibitors targeting the HIF‐α,β complex. By exploiting binding pockets revealed from structural analyses, small‐molecule modulators have the potential to enable selective inhibition of HIF isoforms, and hence control the expression of HIF target genes. In addition to the compounds described above, which target HIF dimerisation, small molecules have been developed to block the HIF:HRE interaction by binding directly to DNA in a sequence‐specific fashion.^91^ Though beyond the scope of this review, this work demonstrates that targeting the HRE is a feasible strategy for HIF inhibition. In the longer term, developing molecules that target defined sets of HIF target genes by selectively binding to HIFs/HREs associated with the promoter regions of specific genes is of interest, though whether or not this is viable is presently unknown.

### 3.3 Targeting CBP/p300

One strategy for HIF inhibition is to block the interaction between HIF‐α and CBP/p300 transcriptional coactivators. CBP and p300 are multi‐domain proteins and present multiple therapeutic possibilities, including inhibition of their bromodomain and acetyltransferase domains.^92^ Here we focus on attempts to block the interaction of the CBP/p300 CH1 domain with the HIF‐α CAD. Pioneering work by Kung et al. led to the development of a high throughput screen for molecules blocking the HIF‐α CAD:p300 CH1 domain interaction.^84^ This work identified the epidithiodiketopiperazine natural product Chetomin (Figure 5) as a disruptor of the HIF:CBP/p300 interaction. Chetomin was observed to inhibit HIF‐mediated transcription in tumour cells,^84^ and likely works by ‘ejecting’ zinc from the p300/CBP CH1 domain; loss of zinc disrupts the CH1 fold and hence binding to the HIF‐1α CAD.^93^ Related mechanisms of action (i.e., zinc ejection/binding) likely apply to the mode of action of other epidithiodiketopiperazines^94^ and may account for their toxic effect to ruminants, including sheep.^93^, ^95^ A high‐throughput screen of natural products also led to the identification of quinones and indandiones that cause loss of structural zinc from CH1.^86^ Eudistidine A, a marine alkaloid with an unusual tetracycline core comprised of two fused pyrimidine and imidazole rings (Figure 5) has also been reported to inhibit the CH1 CAD interaction.^85^

Another strategy used to target the HIF:CBP/p300 interaction has been the development of compounds that mimic the conformation of the HIF‐α CAD when bound to the CH1 domain. Support for this approach comes from observations that overexpression of CAD polypeptides attenuates HIF transcriptional activity in cells.^96^ Two helices that are induced in HIF‐1α upon binding to CH1 have been a focus for development of peptidomimetic inhibitors (Figure 4 b). A number of different scaffolds have been used, including hydrogen bond surrogate helices,^88^, ^97^ aromatic oligoamides,^87^ and oxopiperazine helix mimetics^55^ (Figure 5). These compounds are predicted to bind in an orientation that positions key side‐chains in a manner identical to that observed for the native HIF‐1α helices in the CAD:CH1 structure. For example, peptidomimetics based on Helix 2 mimic the orientation of residues 815–823,^55^, ^87^, ^88^ including two conserved leucine residues (L818 and L822 in HIF‐1α) that bind in a hydrophobic pocket in CBP/p300 (Figure 4 b). Phage display‐based analyses suggest that Helix 2 binds p300 with higher affinity than any other region of the HIF‐1α CAD,^98^ which could explain why most of the reported peptidomimetic compounds have targeted this helix.

The most promising compounds identified to date bind p300 with sub‐micromolar affinities, although the *K*_d_ values are still an order of magnitude higher than those measured for HIF‐1α CAD.^55^, ^88^, ^97^ These compounds are reported to exhibit low cytotoxicity, to be active in down‐regulating HIF target gene expression in cells, and to suppress tumour growth in mouse xenograft models.^55^, ^88^, ^97^ Similar effects on HIF signalling were observed with the inhibitor KCN1, which arose from structure activity relationship studies on sulfonamides blocking HIF:CBP binding.^83^, ^99^ Molecular docking studies suggest that KCN1 may bind at one (or both) of the HIF‐1α helix interaction sites in the HIF/p300 complex.^100^ Peptides that bind to CH1 and displace the HIF‐1α CAD have also been identified by phage‐display methods;^98^ the results of this work may be of interest with respect to developing potent non‐peptide based inhibitors.

### 3.4 Targeting HIF‐α–pVHL Interactions

As outlined above, the binding of prolyl‐hydroxylated HIF‐α to pVHL (so signalling for HIF degradation) is central to hypoxia sensing by the HIF system. Thus, one strategy to mediate upregulation of HIF target genes is to disrupt the HIF‐α:pVHL interaction. Pioneering studies using NODD/CODD peptides indicated that such an approach may be viable.^101^ Work in recent years has focused on testing the viability of pVHL as a target for small molecules blocking HIF‐α degradation, though it should be noted that pVHL likely has roles outside of the HIF system.^102^ Interestingly, this work led to the idea of targeting proteins for degradation by the ubiquitin‐proteasome system, by linking a pVHL binder to a small molecule targeting the protein of interest; this PROTAC (Proteolysis targeting chimeric molecule) strategy holds promise for research as well as therapeutic purposes.^71^ Recently, Bondeson et al. have reported on substantially improved PROTACs, which were shown to have potent activity in cells against the serine‐threonine kinase, RIPK2.^103^

The first non‐peptidic small molecules targeting the HIF‐α:pVHL interaction were designed using computational methods to mimic the structure of the HIF‐1α peptide bound to pVHL.^104^ These compounds recapitulate key interactions in the HIF‐α:pVHL complex, incorporating a *trans*‐4‐hydroxyproline residue, which is crucial for binding to pVHL, as well as an isoxazole moiety designed to interact with a structural water molecule at the HIF‐α:pVHL interface (Figure 3 c). The lead compound from this early work bound to pVHL with a *K*_d_ of 5.4 μm, as determined by isothermal titration calorimetry.^104^, ^105^ Structure activity relationship studies were carried out to increase the binding affinity to pVHL, resulting in a series of compounds that bind pVHL with sub‐micromolar affinity (e.g., Compound **51**, Figure 5).^36^ Further optimisation was carried out using a structure‐ and metrics‐driven approach to increase binding affinity and lipophilicity, with a view to obtaining tight‐binding cell‐active chemical probes.^82^ The most potent inhibitor of the HIF‐α:pVHL interaction identified to date (Compound **7**, Figure 5) binds isolated pVHL with a *K*_d_ of 185 nm, similar to the value observed for a 10‐mer HIF‐1α peptide binding to pVHL (*K*_d_=200 nm).^82^ Although compounds in this series still contain a *trans‐*4‐hydroxyproline, they are largely non‐peptide based. Further work could be targeted towards the development of completely non‐peptidic compounds based on the 4‐hydroxyproline analogues.

## 4 Summary and Outlook

Work over the last 15 years or so has led to the discovery that specific protein–protein interactions play central roles in hypoxic sensing in humans and other animals. Considerable progress has been made towards developing chemically useful inhibitors of the sets of enzymes underlying HIF‐α hydroxylation, that is, the PHDs, and in demonstrating that disrupting the interactions between prolyl hydroxylated HIF‐α and pVHL is a tractable target for small molecules. There remains considerable scope for the development of new types of PHD inhibitor and other compounds that bind to pVHL; in the latter case, the identification of compounds that do not contain a hydroxyproline residue is of interest. It may also be that new challenges in pharmaceutical targeting of pVHL will become apparent as compounds are progressed into animal models, especially due to its HIF‐independent roles. Interestingly, this work has helped stimulate the use of small molecules to rationally target proteins for ‘catalytic’ degradation of protein targets rather than just inhibiting by tight binding. The HIF‐α:FIH and CBP/p300 interactions are seemingly even more challenging due to the apparently HIF‐independent roles of FIH and, in particular, of CBP/p300, which are involved in the regulation of many genes unrelated to HIF. Finally, although at an early stage, use of biophysical insights to guide the development of compounds that bind to and regulate the activity of the intact HIF:HRE complex in order to alter the kinetics of transcription is a particularly exciting field, especially in light of recent structural information.

## Biographical Information

Sarah Wilkins completed her undergraduate and postgraduate studies at the University of Adelaide in Australia. She received a PhD in biochemistry in 2012, and has since been conducting postdoctoral research at the University of Oxford in the laboratory of Professor Schofield. Her research interests include structural and biochemical characterisation of enzymes involved in cellular oxygen‐sensing mechanisms, especially 2‐oxoglutarate‐dependent oxygenases that catalyse protein hydroxylation.

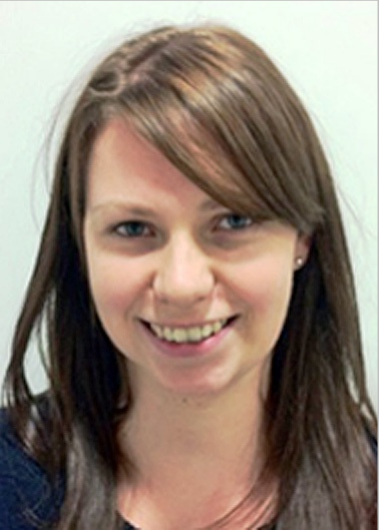

## Biographical Information

Martine Abboud graduated with a BSc in biology and a minor in chemistry from the Lebanese American University. In 2013 she came to Oxford University as a Biochemical Society Krebs Memorial Scholar to pursue a PhD in chemical biology under the supervision of Professor Schofield. Her research foci involve biophysical investigations of protein–protein/ligand interactions, with particular emphasis on 2‐oxoglutarate‐dependent oxygenases and metallo‐β‐lactamases, using various methodologies, in particular, high‐field NMR.

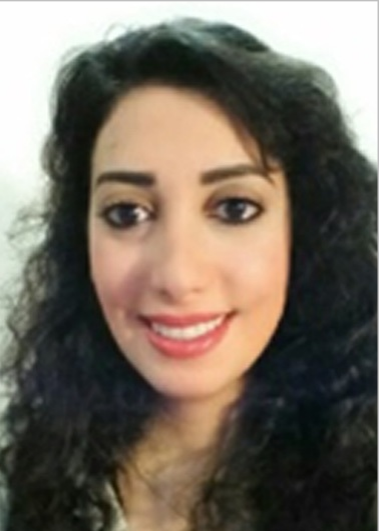

## Biographical Information

Rebecca Hancock graduated with an MChem in chemistry from St. Hilda′s College, Oxford, in 2012. She has since been pursuing a DPhil in cardiovascular medicinal chemistry under the supervision of Dr. Emily Flashman, Dr. Akane Kawamura, and Professor Schofield. Her research interests span hypoxia, epigenetics and cardiovascular disease, and she works between the Departments of Chemistry and Cardiovascular Medicine at Oxford. Alongside her studies, Rebecca edits the Oxford University Biochemical Society magazine, *Phenotype*.

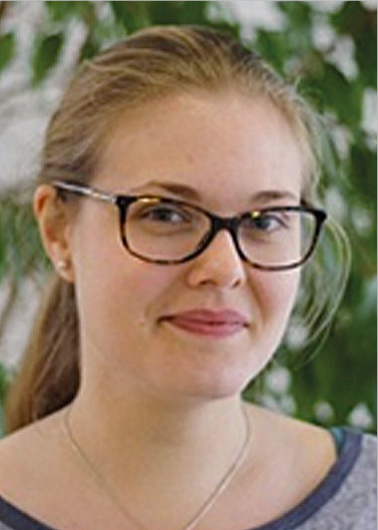

## Biographical Information

Christopher Schofield studied for a first degree in chemistry at the University of Manchester Institute of Science and Technology (1979–1982). In 1982 he moved to Oxford to pursue a PhD with Professor Jack Baldwin on the synthesis and biosynthesis of antibiotics. In 1985 he became a Departmental Demonstrator in the Dyson Perrins Laboratory, Oxford University, followed by his appointment as Lecturer in Chemistry and Fellow of Hertford College in 1990. In 1998 he became Professor of Chemistry, and in 2011 was appointed Head of Organic Chemistry. He is a Fellow of the Royal Society of Chemistry and of the Royal Society. His research group works at the interface of chemistry, biology and medicine. His work has opened up new fields in antibiotic research, oxygen sensing and gene regulation. His work has identified new opportunities for medicinal intervention that are being pursued by academic and commercial laboratories.

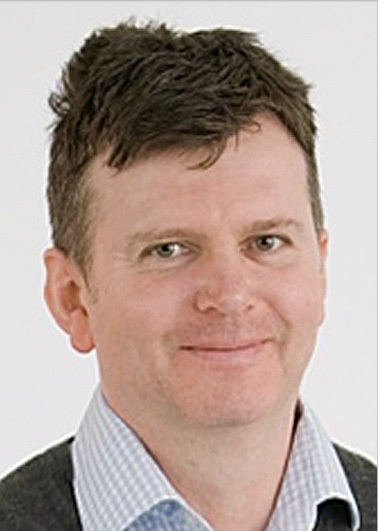

## References

[1] G. L. Semenza , J. Appl. Physiol. 2000, 88, 1474–1480. 10749844 10.1152/jappl.2000.88.4.1474

[2a] G. L. Semenza , Sci. Signaling 2007, 407, cm8;

[2b] A. Weidemann , R. S. Johnson , Cell Death Differ. 2008, 15, 621–627. 18259201 10.1038/cdd.2008.12

[3] C. J. Schofield , P. J. Ratcliffe , Biochem. Biophys. Res. Commun. 2005, 338, 617–626. 16139242 10.1016/j.bbrc.2005.08.111

[4] G. L. Wang , B. Jiang , E. A. Rue , G. L. Semenza , Proc. Natl. Acad. Sci. USA 1995, 92, 5510–5514. 7539918 10.1073/pnas.92.12.5510 PMC41725

[5a] A. Loboda , A. Jozkowicz , J. Dulak , Mol. Cells 2010, 29, 435–442; 20396958 10.1007/s10059-010-0067-2

[5b] P. J. Ratcliffe , J. Clin. Invest. 2007, 117, 862–865. 17404612 10.1172/JCI31750 PMC1838952

[6] B. H. Jiang , J. Z. Zheng , S. W. Leung , R. Roe , G. L. Semenza , J. Biol. Chem. 1997, 272, 19253–19260. 9235919 10.1074/jbc.272.31.19253

[7] L. E. Huang , J. Gu , M. Schau , H. F. Bunn , Proc. Natl. Acad. Sci. USA 1998, 95, 7987–7992. 9653127 10.1073/pnas.95.14.7987 PMC20916

[8] S. Salceda , J. Caro , J. Biol. Chem. 1997, 272, 22642–22647. 9278421 10.1074/jbc.272.36.22642

[9] N. Masson , C. Willam , P. H. Maxwell , C. W. Pugh , P. J. Ratcliffe , EMBO J. 2001, 20, 5197–5206. 11566883 10.1093/emboj/20.18.5197 PMC125617

[10] J. Schödel , S. Grampp , E. R. Maher , H. Moch , P. J. Ratcliffe , P. Russo , D. R. Mole , Eur. Urol. 2016, 69, 646–657. 26298207 10.1016/j.eururo.2015.08.007 PMC5012644

[11a] R. K. Bruick , S. L. McKnight , Science 2001, 294, 1337–1340; 11598268 10.1126/science.1066373

[11b] A. C. R. Epstein , J. M. Gleadle , L. A. McNeill , K. S. Hewitson , J. O′Rourke , D. R. Mole , M. Mukherji , E. Metzen , M. I. Wilson , A. Dhanda , Y.-M. Tian , N. Masson , D. L. Hamilton , P. Jaakkola , R. Barstead , J. Hodgkin , P. H. Maxwell , C. W. Pugh , C. J. Schofield , P. J. Ratcliffe , Cell 2001, 107, 43–54; 11595184 10.1016/s0092-8674(01)00507-4

[11c] M. Ivan , T. Haberberger , D. C. Gervasi , K. S. Michelson , V. Günzler , K. Kondo , H. Yang , I. Sorokina , R. C. Conaway , J. W. Conaway , W. G. Kaelin , Proc. Natl. Acad. Sci. USA 2002, 99, 13459–13464; 12351678 10.1073/pnas.192342099 PMC129695

[11d] P. Jaakkola , D. R. Mole , Y. M. Tian , M. I. Wilson , J. Gielbert , S. J. Gaskell , A. von Kriegsheim , H. F. Hebestreit , M. Mukherji , C. J. Schofield , P. H. Maxwell , C. W. Pugh , P. J. Ratcliffe , Science 2001, 292, 468–472. 11292861 10.1126/science.1059796

[12a] E. Berra , E. Benizri , A. Ginouves , V. Volmat , D. Roux , J. Pouyssegur , EMBO J. 2003, 22, 4082–4090; 12912907 10.1093/emboj/cdg392 PMC175782

[12b] J. H. Dao , R. J. M. Kurzeja , J. M. Morachis , H. Veith , J. Lewis , V. Yu , C. M. Tegley , P. Tagari , Anal. Biochem. 2009, 384, 213–223; 18952043 10.1016/j.ab.2008.09.052

[12c] D. Ehrismann , E. Flashman , D. N. Genn , N. Mathioudakis , K. S. Hewitson , P. J. Ratcliffe , C. J. Schofield , Biochem. J. 2007, 401, 227–234; 16952279 10.1042/BJ20061151 PMC1698668

[12d] M. Hirsilä , P. Koivunen , V. Gunzler , K. I. Kivirikko , J. Myllyharju , J. Biol. Chem. 2003, 278, 30772–30780; 12788921 10.1074/jbc.M304982200

[12e] H. Tarhonskaya , R. Chowdhury , I. K. Leung , N. D. Loik , J. S. McCullagh , T. D. Claridge , C. J. Schofield , E. Flashman , Biochem. J. 2014, 463, 363–372; 25120187 10.1042/BJ20140779

[12f] Y. M. Tian , K. K. Yeoh , M. K. Lee , T. Eriksson , B. M. Kessler , H. B. Kramer , M. J. Edelmann , C. Willam , C. W. Pugh , C. J. Schofield , P. J. Ratcliffe , J. Biol. Chem. 2011, 286, 13041–13051. 21335549 10.1074/jbc.M110.211110 PMC3075650

[13] W. C. Hon , M. I. Wilson , K. Harlos , T. D. W. Claridge , C. J. Schofield , C. W. Pugh , P. H. Maxwell , P. J. Ratcliffe , D. I. Stuart , E. Y. Jones , Nature 2002, 417, 975–978. 12050673 10.1038/nature00767

[14] S. Martinez , R. P. Hausinger , J. Biol. Chem. 2015, 290, 20702–20711. 26152721 10.1074/jbc.R115.648691 PMC4543632

[15] E. Flashman , L. M. Hoffart , R. B. Hamed , J. M. Bollinger, Jr. , C. Krebs , C. J. Schofield , FEBS J. 2010, 277, 4089–4099. 20840591 10.1111/j.1742-4658.2010.07804.x PMC4160827

[16a] K. S. Hewitson , L. A. McNeill , M. V. Riordan , Y. M. Tian , A. N. Bullock , R. W. D. Welford , J. M. Elkins , N. J. Oldham , S. Battacharya , J. Gleadle , P. J. Ratcliffe , C. W. Pugh , C. J. Schofield , J. Biol. Chem. 2002, 277, 26351–26355; 12042299 10.1074/jbc.C200273200

[16b] D. Lando , D. J. Peet , J. J. Gorman , D. A. Whelan , M. L. Whitelaw , R. K. Bruick , Genes Dev. 2002, 16, 1466–1471. 12080085 10.1101/gad.991402 PMC186346

[17] D. Lando , D. J. Peet , D. A. Whelan , J. J. Gorman , M. L. Whitelaw , Science 2002, 295, 858–861. 11823643 10.1126/science.1068592

[18a] Z. Arany , L. E. Huang , R. Eckner , S. Bhattacharya , C. Jiang , M. A. Goldberg , H. F. Bunn , D. M. Livingston , Proc. Natl. Acad. Sci. USA 1996, 93, 12969–12973; 8917528 10.1073/pnas.93.23.12969 PMC24030

[18b] M. Ema , K. Hirota , J. Mimura , H. Abe , J. Yodoi , K. Sogawa , L. Poellinger , Y. Fujii-Kuriyama , EMBO J. 1999, 18, 1905–1914; 10202154 10.1093/emboj/18.7.1905 PMC1171276

[18c] P. J. Kallio , K. Okamoto , S. O′Brien , P. Carrero , Y. Makino , H. Tanaka , L. Poellinger , EMBO J. 1998, 17, 6573–6586; 9822602 10.1093/emboj/17.22.6573 PMC1171004

[18d] B. L. Ebert , H. F. Bunn , Mol. Cell. Biol. 1998, 18, 4089–4096. 9632793 10.1128/mcb.18.7.4089 PMC108993

[19a] P. Koivunen , M. Hirsilä , V. Günzler , K. I. Kivirikko , J. Myllyharju , J. Biol. Chem. 2004, 279, 9899–9904; 14701857 10.1074/jbc.M312254200

[19b] M. B. Pappalardi , D. E. McNulty , J. D. Martin , K. E. Fisher , Y. Jiang , M. C. Burns , H. Zhao , T. Ho , S. Sweitzer , B. Schwartz , R. S. Annan , R. A. Copeland , P. J. Tummino , L. Luo , Biochem. J. 2011, 436, 363–369. 21410436 10.1042/BJ20101201

[20] B. H. Jiang , E. Rue , G. L. Wang , R. Roe , G. L. Semenza , J. Biol. Chem. 1996, 271, 17771–17778. 8663540 10.1074/jbc.271.30.17771

[21] D. L. Wu , N. Potluri , J. P. Lu , Y. C. Kim , F. Rastinejad , Nature 2015, 524, 303–309. 26245371 10.1038/nature14883

[22] A. Chapman-Smith , J. K. Lutwyche , M. L. Whitelaw , J. Biol. Chem. 2004, 279, 5353–5362. 14638687 10.1074/jbc.M310041200

[23a] H. Tian , S. L. McKnight , D. W. Russell , Genes Dev. 1997, 11, 72–82; 9000051 10.1101/gad.11.1.72

[23b] M. Ema , S. Taya , N. Yokotani , K. Sogawa , Y. Matsuda , Y. Fujii-Kuriyama , Proc. Natl. Acad. Sci. USA 1997, 94, 4273–4278; 9113979 10.1073/pnas.94.9.4273 PMC20712

[23c] M. S. Wiesener , H. Turley , W. E. Allen , C. Willam , K. U. Eckardt , K. L. Talks , S. M. Wood , K. C. Gatter , A. L. Harris , C. W. Pugh , P. J. Ratcliffe , P. H. Maxwell , Blood 1998, 92, 2260–2268. 9746763

[24] J. H. Min , H. F. Yang , M. Ivan , F. Gertler , W. G. Kaelin , N. P. Pavletich , Science 2002, 296, 1886–1889. 12004076 10.1126/science.1073440

[25] F. Miller , A. Kentsis , R. Osman , Z. Q. Pan , J. Biol. Chem. 2005, 280, 7985–7996. 15611064 10.1074/jbc.M413160200

[26] C. J. Illingworth , C. Loenarz , C. J. Schofield , C. Domene , Biochemistry 2010, 49, 6936–6944. 20695530 10.1021/bi100358t

[27] R. Chowdhury , M. A. McDonough , J. Mecinovic , C. Loenarz , E. Flashman , K. S. Hewitson , C. Domene , C. J. Schofield , Structure 2009, 17, 981–989. 19604478 10.1016/j.str.2009.06.002

[28] E. Flashman , E. A. L. Bagg , R. Chowdhury , J. Mecinovic , C. Loenarz , M. A. McDonough , K. S. Hewitson , C. J. Schofield , J. Biol. Chem. 2008, 283, 3808–3815. 18063574 10.1074/jbc.M707411200

[29] M. A. McDonough , V. Li , E. Flashman , R. Chowdhury , C. Mohr , B. M. Lienard , J. Zondlo , N. J. Oldham , I. J. Clifton , J. Lewis , L. A. McNeill , R. J. Kurzeja , K. S. Hewitson , E. Yang , S. Jordan , R. S. Syed , C. J. Schofield , Proc. Natl. Acad. Sci. USA 2006, 103, 9814–9819. 16782814 10.1073/pnas.0601283103 PMC1502536

[30] J. S. Scotti , I. K. H. Leung , W. Ge , M. A. Bentley , J. Paps , H. B. Kramer , J. Lee , W. Aik , H. Choi , S. M. Paulsen , L. A. H. Bowman , N. D. Loik , S. Horita , C.-h. Ho , N. J. Kershaw , C. M. Tang , T. D. W. Claridge , G. M. Preston , M. A. McDonough , C. J. Schofield , Proc. Natl. Acad. Sci. USA 2014, 111, 13331–13336. 25197067 10.1073/pnas.1409916111 PMC4169948

[31] Y. Kageyama , M. Koshiji , K. K. To , Y. M. Tian , P. J. Ratcliffe , L. E. Huang , FASEB J. 2004, 18, 1028–1030. 15084514 10.1096/fj.03-1233fje

[32] C. E. Snell , H. Turley , A. McIntyre , D. Li , M. Masiero , C. J. Schofield , K. C. Gatter , A. L. Harris , F. Pezzella , PLoS ONE 2014, 9, e88955. 10.1371/journal.pone.0088955 PMC3923075 24563687

[33a] K. Gradin , J. McGuire , R. H. Wenger , I. Kvietikova , M. L. Whitelaw , R. Toftgard , L. Tora , M. Gassmann , L. Poellinger , Mol. Cell. Biol. 1996, 16, 5221–5231; 8816435 10.1128/mcb.16.10.5221 PMC231522

[33b] W. Luo , J. Zhong , R. Chang , H. Hu , A. Pandey , G. L. Semenza , J. Biol. Chem. 2010, 285, 3651–3663. 19940151 10.1074/jbc.M109.068577 PMC2823506

[34] D. M. Katschinski , L. Le , S. G. Schindler , T. Thomas , A. K. Voss , R. H. Wenger , Cell. Physiol. Biochem. 2004, 14, 351–360. 15319539 10.1159/000080345

[35] Y. V. Liu , J. H. Baek , H. Zhang , R. Diez , R. N. Cole , G. L. Semenza , Mol. Cell 2007, 25, 207–217. 17244529 10.1016/j.molcel.2007.01.001 PMC2563152

[36] D. L. Buckley , J. L. Gustafson , I. Van Molle , A. G. Roth , H. S. Tae , P. C. Gareiss , W. L. Jorgensen , A. Ciulli , C. M. Crews , Angew. Chem. Int. Ed. 2012, 51, 11463–11467; 10.1002/anie.201206231 PMC3519281 23065727

[37] M. C. Chan , O. Atasoylu , E. Hodson , A. Tumber , I. K. Leung , R. Chowdhury , V. Gomez-Perez , M. Demetriades , A. M. Rydzik , J. Holt-Martyn , Y. M. Tian , T. Bishop , T. D. Claridge , A. Kawamura , C. W. Pugh , P. J. Ratcliffe , C. J. Schofield , PLoS ONE 2015, 10, e0132004. 10.1371/journal.pone.0132004 PMC4492579 26147748

[38] D. Song , L. S. Li , K. J. Heaton-Johnson , P. R. Arsenault , S. R. Master , F. S. Lee , J. Biol. Chem. 2013, 288, 9662–9674. 23413029 10.1074/jbc.M112.440552 PMC3617269

[39] D. E. Foxler , K. S. Bridge , V. James , T. M. Webb , M. Mee , S. C. Wong , Y. Feng , D. Constantin-Teodosiu , T. E. Petursdottir , J. Bjornsson , S. Ingvarsson , P. J. Ratcliffe , G. D. Longmore , T. V. Sharp , Nat. Cell Biol. 2012, 14, 201–208. 22286099 10.1038/ncb2424

[40] B. W. Wong , A. Kuchnio , U. Bruning , P. Carmeliet , Trends Biochem. Sci. 2013, 38, 3–11. 23200187 10.1016/j.tibs.2012.10.004

[41] J. L. Ruas , U. Berchner-Pfannschmidt , S. Malik , K. Gradin , J. Fandrey , R. G. Roeder , T. Pereira , L. Poellinger , J. Biol. Chem. 2010, 285, 2601–2609. 19880525 10.1074/jbc.M109.021824 PMC2807317

[42a] S. J. Freedman , Z.-Y. J. Sun , F. Poy , A. L. Kung , D. M. Livingston , G. Wagner , M. J. Eck , Proc. Natl. Acad. Sci. USA 2002, 99, 5367–5372; 11959990 10.1073/pnas.082117899 PMC122775

[42b] S. A. Dames , M. Martinez-Yamout , R. N. De Guzman , H. J. Dyson , P. E. Wright , Proc. Natl. Acad. Sci. USA 2002, 99, 5271–5276. 11959977 10.1073/pnas.082121399 PMC122759

[43] L. A. McNeill , K. S. Hewitson , T. D. W. Claridge , J. F. Seibel , L. E. Horsfall , C. J. Schofield , Biochem. J. 2002, 367, 571–575. 12215170 10.1042/BJ20021162 PMC1222951

[44] J. M. Elkins , K. S. Hewitson , L. A. McNeill , J. F. Seibel , I. Schlemminger , C. W. Pugh , P. J. Ratcliffe , C. J. Schofield , J. Biol. Chem. 2003, 278, 1802–1806. 12446723 10.1074/jbc.C200644200

[45a] M. L. Coleman , P. J. Ratcliffe , Curr. Pharm. Des. 2009, 15, 3904–3907; 19671041 10.2174/138161209789649448

[45b] M. E. Cockman , J. D. Webb , P. J. Ratcliffe , Ann N Y Acad Sci 2009, 1177, 9–18. 19845602 10.1111/j.1749-6632.2009.05042.x

[46a] A. P. Hardy , I. Prokes , L. Kelly , I. D. Campbell , C. J. Schofield , J Mol Biol 2009, 392, 994–1006; 19646994 10.1016/j.jmb.2009.07.070

[46b] S. E. Wilkins , S. Karttunen , R. J. Hampton-Smith , I. Murchland , A. Chapman-Smith , D. J. Peet , J. Biol. Chem. 2012, 287, 8769–8781. 22270367 10.1074/jbc.M111.294678 PMC3308777

[47a] M. Yang , R. Chowdhury , W. Ge , R. B. Hamed , M. A. McDonough , T. D. Claridge , B. M. Kessler , M. E. Cockman , P. J. Ratcliffe , C. J. Schofield , FEBS J. 2011, 278, 1086–1097; 21251231 10.1111/j.1742-4658.2011.08022.x PMC3569879

[47b] M. Yang , W. Ge , R. Chowdhury , T. D. Claridge , H. B. Kramer , B. Schmierer , M. A. McDonough , L. Gong , B. M. Kessler , P. J. Ratcliffe , M. L. Coleman , C. J. Schofield , J. Biol. Chem. 2011, 286, 7648–7660. 21177872 10.1074/jbc.M110.193540 PMC3045019

[48a] M. L. Coleman , M. A. McDonough , K. S. Hewitson , C. Coles , J. Mecinovic , M. Edelmann , K. M. Cook , M. E. Cockman , D. E. Lancaster , B. M. Kessler , N. J. Oldham , P. J. Ratcliffe , C. J. Schofield , J. Biol. Chem. 2007, 282, 24027–24038; 17573339 10.1074/jbc.M704102200

[48b] X. Zheng , S. Linke , J. M. Dias , X. Zheng , K. Gradin , T. P. Wallis , B. R. Hamilton , M. Gustafsson , J. L. Ruas , S. Wilkins , R. L. Bilton , K. Brismar , M. L. Whitelaw , T. Pereira , J. J. Gorman , J. Ericson , D. J. Peet , U. Lendahl , L. Poellinger , Proc. Natl. Acad. Sci. USA 2008, 105, 3368–3373. 18299578 10.1073/pnas.0711591105 PMC2265116

[49] L. Kelly , M. A. McDonough , M. L. Coleman , P. J. Ratcliffe , C. J. Schofield , Mol. BioSyst. 2009, 5, 52–58. 19081931 10.1039/b815271c

[50] R. S. Singleton , D. C. Trudgian , R. Fischer , B. M. Kessler , P. J. Ratcliffe , M. E. Cockman , J. Biol. Chem. 2011, 286, 33784–33794. 21808058 10.1074/jbc.M111.262808 PMC3190818

[51] R. A. Berg , D. J. Prockop , Biochem. Biophys. Res. Commun. 1973, 52, 115–120. 4712181 10.1016/0006-291x(73)90961-3

[52] J. D. Webb , A. Muranyi , C. W. Pugh , P. J. Ratcliffe , M. L. Coleman , Biochem. J. 2009, 420, 327–333. 19245366 10.1042/BJ20081905

[53] B. Schmierer , B. Novak , C. J. Schofield , BMC Syst. Biol. 2010, 4, 139. 20955552 10.1186/1752-0509-4-139 PMC2984394

[54] M. A. McDonough , L. A. McNeill , M. Tilliet , C. A. Papamicael , Q. Y. Chen , B. Banerji , K. S. Hewitson , C. J. Schofield , J. Am. Chem. Soc. 2005, 127, 7680–7681. 15913349 10.1021/ja050841b

[55] B. B. Lao , I. Grishagin , H. Mesallati , T. F. Brewer , B. Z. Olenyuk , P. S. Arora , Proc. Natl. Acad. Sci. USA 2014, 111, 7531–7536. 24821806 10.1073/pnas.1402393111 PMC4040591

[56] M. V. Gustafsson , X. Zheng , T. Pereira , K. Gradin , S. Jin , J. Lundkvist , J. L. Ruas , L. Poellinger , U. Lendahl , M. Bondesson , Dev Cell 2005, 9, 617–628. 16256737 10.1016/j.devcel.2005.09.010

[57] P. van Uden , N. S. Kenneth , S. Rocha , Biochem. J. 2008, 412, 477–484. 18393939 10.1042/BJ20080476 PMC2474706

[58a] V. L. Dengler , M. D. Galbraith , J. M. Espinosa , Crit. Rev. Biochem. Mol. Biol. 2014, 49, 1–15; 24099156 10.3109/10409238.2013.838205 PMC4342852

[58b] A. Melvin , S. Rocha , Cell. Signalling 2012, 24, 35–43. 21924352 10.1016/j.cellsig.2011.08.019 PMC3476533

[59a] R. J. Klose , E. M. Kallin , Y. Zhang , Nat. Rev. Genet. 2006, 7, 715–727; 16983801 10.1038/nrg1945

[59b] A. J. Krieg , E. B. Rankin , D. Chan , O. Razorenova , S. Fernandez , A. J. Giaccia , Mol. Cell. Biol. 2010, 30, 344–353; 19858293 10.1128/MCB.00444-09 PMC2798291

[59c] R. L. Hancock , K. Dunne , L. J. Walport , E. Flashman , A. Kawamura , Epigenomics 2015, 7, 791–811. 25832587 10.2217/epi.15.24

[60] R. J. Kewley , M. L. Whitelaw , A. Chapman-Smith , Int. J. Biochem. Cell Biol. 2004, 36, 189–204. 14643885 10.1016/s1357-2725(03)00211-5

[61] Y. Makino , R. Cao , K. Svensson , G. Bertilsson , M. Asman , H. Tanaka , Y. Cao , A. Berkenstam , L. Poellinger , Nature 2001, 414, 550–554. 11734856 10.1038/35107085

[62] T. V. Beischlag , R. T. Taylor , D. W. Rose , D. Yoon , Y. M. Chen , W. H. Lee , M. G. Rosenfeld , O. Hankinson , J. Biol. Chem. 2004, 279, 54620–54628. 15485806 10.1074/jbc.M410456200

[63] Y. Guo , C. L. Partch , J. Key , P. B. Card , V. Pashkov , A. Patel , R. K. Bruick , H. Wurdak , K. H. Gardner , ACS Chem. Biol. 2013, 8, 626–635. 23240775 10.1021/cb300604u PMC3600089

[64] G. L. Wang , G. L. Semenza , Proc. Natl. Acad. Sci. USA 1993, 90, 4304–4308. 8387214 10.1073/pnas.90.9.4304 PMC46495

[65a] X. Q. Guo , Z. T. Tian , X. L. Wang , S. H. Pan , W. R. Huang , Y. Q. Shen , Y. T. Gui , X. L. Duan , Z. M. Cai , Acta Biochim. Biophys. Sin. 2015, 47, 106–113; 25520177 10.1093/abbs/gmu122

[65b] W. B. Luo , R. Chang , J. Zhong , A. Pandey , G. L. Semenza , Proc. Natl. Acad. Sci. USA 2012, 109, E3367–E3376. 10.1073/pnas.1217394109 PMC3523832 23129632

[66] A. S. Bhagwat , C. R. Vakoc , Trends Cancer 2015, 1, 53–65. 26645049 10.1016/j.trecan.2015.07.001 PMC4669894

[67] M. Burtscher , Aging Dis. 2014, 5, 274–280. 25110611 10.14336/AD.2014.0500274 PMC4113517

[68a] L. Berk , J. H. Burchenal , W. B. Castle , New Engl. J. Med. 1949, 240, 754–761; 18120180 10.1056/NEJM194905122401903

[68b] J. Wolf , I. J. Levy , Arch. Intern. Med. 1954, 93, 387–396. 10.1001/archinte.1954.00240270073007 13123563

[69a] M. C. Chan , J. P. Holt-Martyn , C. J. Schofield , P. J. Ratcliffe , Mol. Aspects Med. 2016, 47–48, 54–75; 10.1016/j.mam.2016.01.001 26791432

[69b] P. H. Maxwell , K. U. Eckardt , Nat. Rev. Nephrol. 2015, 12, 157–168. 26656456 10.1038/nrneph.2015.193

[70a] G. N. Masoud , W. Li , Acta Pharm. Sin. B 2015, 5, 378–389; 26579469 10.1016/j.apsb.2015.05.007 PMC4629436

[70b] G. Melillo , Methods Enzymol. 2007, 435, 385–402. 17998065 10.1016/S0076-6879(07)35020-9

[71] P. Bargagna-Mohan , S. H. Baek , H. Lee , K. Kim , R. Mohan , Bioorg. Med. Chem. Lett. 2005, 15, 2724–2727. 15876533 10.1016/j.bmcl.2005.04.008 PMC3226786

[72] R. R. Raval , K. W. Lau , M. G. B. Tran , H. M. Sowter , S. J. Mandriota , J.-L. Li , C. W. Pugh , P. H. Maxwell , A. L. Harris , P. J. Ratcliffe , Mol. Cell. Biol. 2005, 25, 5675–5686. 15964822 10.1128/MCB.25.13.5675-5686.2005 PMC1157001

[73] K. K. Kim , S. Abelman , N. Yano , J. R. Ribeiro , R. K. Singh , M. Tipping , R. G. Moore , Sci. Rep. 2015, 5, 14296. 26469226 10.1038/srep14296 PMC4606568

[74] R. Myllylä , E. R. Kuutti-Savolainen , K. I. Kivirikko , Biochem. Biophys. Res. Commun. 1978, 83, 441–448. 212056 10.1016/0006-291x(78)91010-0

[75] J. Pouysségur , F. Mechta-Grigoriou , Biol Chem. 2006, 387, 1337–1346. 17081104 10.1515/BC.2006.167

[76] N. R. Rose , M. A. McDonough , O. N. F. King , A. Kawamura , C. J. Schofield , Chem. Soc. Rev. 2011, 40, 4364–4397. 21390379 10.1039/c0cs00203h

[77] S. Salceda , I. Beck , J. Caro , Arch. Biochem. Biophys. 1996, 334, 389–394. 8900415 10.1006/abbi.1996.0469

[78] K. Lee , H. Zhang , D. Z. Qian , S. Rey , J. O. Liu , G. L. Semenza , Proc. Natl. Acad. Sci. USA 2009, 106, 17910–17915. 19805192 10.1073/pnas.0909353106 PMC2764905

[79a] C. J. Lee , C. H. Yue , Y. J. Lin , Y. Y. Lin , S. H. Kao , J. Y. Liu , Y. H. Chen , Anticancer Res. 2014, 34, 6467–6472; 25368247

[79b] C. J. Lee , C. H. Yue , Y. Y. Lin , J. C. Wu , J. Y. Liu , Anticancer Res. 2014, 34, 3549–3556. 24982368

[80a] J. Key , T. H. Scheuermann , P. C. Anderson , V. Daggett , K. H. Gardner , J. Am. Chem. Soc. 2009, 131, 17647–17654; 19950993 10.1021/ja9073062 PMC2819816

[80b] J. L. Rogers , L. Bayeh , T. H. Scheuermann , J. Longgood , J. Key , J. Naidoo , L. Melito , C. Shokri , D. E. Frantz , R. K. Bruick , K. H. Gardner , J. B. MacMillan , U. K. Tambar , J. Med. Chem. 2013, 56, 1739–1747; 23363003 10.1021/jm301847z PMC3676484

[80c] T. H. Scheuermann , Q. Li , H. W. Ma , J. Key , L. Zhang , R. Chen , J. A. Garcia , J. Naidoo , J. Longgood , D. E. Frantz , U. K. Tambar , K. H. Gardner , R. K. Bruick , Nat. Chem. Biol. 2013, 9, 271–276; 23434853 10.1038/nchembio.1185 PMC3604136

[80d] T. H. Scheuermann , D. R. Tomchick , M. Machius , Y. Guo , R. K. Bruick , K. H. Gardner , Proc. Natl. Acad. Sci. USA 2009, 106, 450–455. 19129502 10.1073/pnas.0808092106 PMC2626723

[81] E. Miranda , I. K. Nordgren , A. L. Male , C. E. Lawrence , F. Hoakwie , F. Cuda , W. Court , K. R. Fox , P. A. Townsend , G. K. Packham , S. A. Eccles , A. Tavassoli , J. Am. Chem. Soc. 2013, 135, 10418–10425. 23796364 10.1021/ja402993u PMC3715890

[82] C. Galdeano , M. S. Gadd , P. Soares , S. Scaffidi , I. Van Molle , I. Birced , S. Hewitt , D. M. Dias , A. Ciulli , J. Med. Chem. 2014, 57, 8657–8663. 25166285 10.1021/jm5011258 PMC4207132

[83] C. Tan , R. G. de Noronha , N. S. Devi , A. A. Jabbar , S. Kaluz , Y. Liu , S. R. Mooring , K. C. Nicolaou , B. Wang , E. G. Van Meir , Bioorg. Med. Chem. Lett. 2011, 21, 5528–5532. 21831638 10.1016/j.bmcl.2011.06.099 PMC3292863

[84] A. L. Kung , S. D. Zabludoff , D. S. France , S. J. Freedman , E. A. Tanner , A. Vieira , S. Cornell-Kennon , J. Lee , B. Wang , J. Wang , K. Memmert , H. U. Naegeli , F. Petersen , M. J. Eck , K. W. Bair , A. W. Wood , D. M. Livingston , Cancer Cell 2004, 6, 33–43. 15261140 10.1016/j.ccr.2004.06.009

[85] S. T. Chan , P. R. Patel , T. R. Ransom , C. J. Henrich , T. C. McKee , A. K. Goey , K. M. Cook , W. D. Figg , J. B. McMahon , M. J. Schnermann , K. R. Gustafson , J. Am. Chem. Soc. 2015, 137, 5569–5575. 25892103 10.1021/jacs.5b02156 PMC6318789

[86] M. K. Jayatunga , S. Thompson , T. C. McKee , M. C. Chan , K. M. Reece , A. P. Hardy , R. Sekirnik , P. T. Seden , K. M. Cook , J. B. McMahon , W. D. Figg , C. J. Schofield , A. D. Hamilton , Eur. J. Med. Chem. 2015, 94, 509–516. 25023609 10.1016/j.ejmech.2014.06.006 PMC4277744

[87] G. M. Burslem , H. F. Kyle , A. L. Breeze , T. A. Edwards , A. Nelson , S. L. Warriner , A. J. Wilson , ChemBioChem 2014, 15, 1083–1087. 24782431 10.1002/cbic.201400009 PMC4159589

[88] S. Kushal , B. B. Lao , L. K. Henchey , R. Dubey , H. Mesallati , N. J. Traaseth , B. Z. Olenyuk , P. S. Arora , Proc. Natl. Acad. Sci. USA 2013, 110, 15602–15607. 24019500 10.1073/pnas.1312473110 PMC3785738

[89] M. Masetti , F. Falchi , M. Recanatini , PLoS ONE 2014, 9, e94986. 10.1371/journal.pone.0094986 PMC3988133 24736273

[90] R. Cardoso , R. Love , C. L. Nilsson , S. Bergqvist , D. Nowlin , J. Yan , K. K. Liu , J. Zhu , P. Chen , Y. L. Deng , H. J. Dyson , M. J. Greig , A. Brooun , Protein Sci. 2012, 21, 1885–1896. 23033253 10.1002/pro.2172 PMC3575918

[91a] D. Kong , E. J. Park , A. G. Stephen , M. Calvani , J. H. Cardellina , A. Monks , R. J. Fisher , R. H. Shoemaker , G. Melillo , Cancer Res. 2005, 65, 9047–9055; 16204079 10.1158/0008-5472.CAN-05-1235

[91b] B. Z. Olenyuk , G. J. Zhang , J. M. Klco , N. G. Nickols , W. G. Kaelin, Jr. , P. B. Dervan , Proc. Natl. Acad. Sci. USA 2004, 101, 16768–16773. 15556999 10.1073/pnas.0407617101 PMC534742

[92a] D. A. Hay , O. Fedorov , S. Martin , D. C. Singleton , C. Tallant , C. Wells , S. Picaud , M. Philpott , O. P. Monteiro , C. M. Rogers , S. J. Conway , T. P. Rooney , A. Tumber , C. Yapp , P. Filippakopoulos , M. E. Bunnage , S. Muller , S. Knapp , C. J. Schofield , P. E. Brennan , J. Am. Chem. Soc. 2014, 136, 9308–9319; 24946055 10.1021/ja412434f PMC4183655

[92b] K. Mantelingu , B. A. Reddy , V. Swaminathan , A. H. Kishore , N. B. Siddappa , G. V. Kumar , G. Nagashankar , N. Natesh , S. Roy , P. P. Sadhale , U. Ranga , C. Narayana , T. K. Kundu , Chem. Biol. 2007, 14, 645–657. 17584612 10.1016/j.chembiol.2007.04.011

[93] K. M. Cook , S. T. Hilton , J. Mecinovic , W. B. Motherwell , W. D. Figg , C. J. Schofield , J. Biol. Chem. 2009, 284, 26831–26838. 19589782 10.1074/jbc.M109.009498 PMC2785371

[94a] R. Dubey , M. D. Levin , L. Z. Szabo , C. F. Laszlo , S. Kushal , J. B. Singh , P. Oh , J. E. Schnitzer , B. Z. Olenyuk , J. Am. Chem. Soc. 2013, 135, 4537–4549; 23448368 10.1021/ja400805b

[94b] F. L. Cherblanc , K. L. Chapman , J. Reid , A. J. Borg , S. Sundriyal , L. Alcazar-Fuoli , E. Bignell , M. Demetriades , C. J. Schofield , P. A. DiMaggio, Jr. , R. Brown , M. J. Fuchter , J. Med. Chem. 2013, 56, 8616–8625. 24099080 10.1021/jm401063r

[95] C. L. Chai , P. Waring , Redox Rep. 2000, 5, 257–264. 11145100 10.1179/135100000101535799

[96] A. L. Kung , S. Wang , J. M. Klco , W. G. Kaelin , D. M. Livingston , Nat. Med. 2000, 6, 1335–1340. 11100117 10.1038/82146

[97] L. K. Henchey , S. Kushal , R. Dubey , R. N. Chapman , B. Z. Olenyuk , P. S. Arora , J. Am. Chem. Soc. 2010, 132, 941–943. 20041650 10.1021/ja9082864 PMC2810346

[98] H. F. Kyle , K. F. Wickson , J. Stott , G. M. Burslem , A. L. Breeze , C. Tiede , D. C. Tomlinson , S. L. Warriner , A. Nelson , A. J. Wilson , T. A. Edwards , Mol. BioSyst. 2015, 11, 2738–2749. 26135796 10.1039/c5mb00284b

[99] S. R. Mooring , H. Jin , N. S. Devi , A. A. Jabbar , S. Kaluz , Y. Liu , E. G. Van Meir , B. Wang , J. Med. Chem. 2011, 54, 8471–8489. 22032632 10.1021/jm201018g PMC3292864

[100] Q. Shi , S. Yin , S. Kaluz , N. Ni , N. S. Devi , J. Mun , D. Wang , K. Damera , W. Chen , S. Burroughs , S. R. Mooring , M. M. Goodman , E. G. Van Meir , B. Wang , J. P. Snyder , ACS Med. Chem. Lett. 2012, 3, 620–625. 24936238 10.1021/ml300042k PMC4056939

[101] C. Willam , N. Masson , Y. M. Tian , S. A. Mahmood , M. I. Wilson , R. Bicknell , K. U. Eckardt , P. H. Maxwell , P. J. Ratcliffe , C. W. Pugh , Proc. Natl. Acad. Sci. USA 2002, 99, 10423–10428. 12149454 10.1073/pnas.162119399 PMC124930

[102] C. M. Robinson , M. Ohh , FEBS Lett. 2014, 588, 2704–2711. 24583008 10.1016/j.febslet.2014.02.026

[103] D. P. Bondeson , A. Mares , I. E. Smith , E. Ko , S. Campos , A. H. Miah , K. E. Mulholland , N. Routly , D. L. Buckley , J. L. Gustafson , N. Zinn , P. Grandi , S. Shimamura , G. Bergamini , M. Faelth-Savitski , M. Bantscheff , C. Cox , D. A. Gordon , R. R. Willard , J. J. Flanagan , L. N. Casillas , B. J. Votta , W. den Besten , K. Famm , L. Kruidenier , P. S. Carter , J. D. Harling , I. Churcher , C. M. Crews , Nat. Chem. Biol. 2015, 11, 611–617. 26075522 10.1038/nchembio.1858 PMC4629852

[104] D. L. Buckley , I. Van Molle , P. C. Gareiss , H. S. Tae , J. Michel , D. J. Noblin , W. L. Jorgensen , A. Ciulli , C. M. Crews , J. Am. Chem. Soc. 2012, 134, 4465–4468. 22369643 10.1021/ja209924v PMC3448299

[105] I. Van Molle , A. Thomann , D. L. Buckley , E. C. So , S. Lang , C. M. Crews , A. Ciulli , Chem. Biol. 2012, 19, 1300–1312. 23102223 10.1016/j.chembiol.2012.08.015 PMC3551621

